# Zyxin‐involved actin regulation is essential in the maintenance of vinculin focal adhesion and chondrocyte differentiation status

**DOI:** 10.1111/cpr.12532

**Published:** 2018-10-17

**Authors:** Gaoming Li, Xiongbo Song, Rui Li, Li Sun, Xiaoyuan Gong, Cheng Chen, Liu Yang

**Affiliations:** ^1^ Key Laboratory of Freshwater Fish Reproduction and Development, Ministry of Education, Laboratory of Molecular Developmental Biology School of Life Sciences, Southwest University Chongqing China; ^2^ Center for Joint Surgery Southwest Hospital, Third Military Medical University (Army Medical University) Chongqing China; ^3^ Department of Orthopedics Guizhou Provincial People's Hospital Guiyang China

## Abstract

**Objectives:**

To investigate the role of zyxin‐involved actin regulation in expression level of vinculin focal adhesion and collagen production of chondrocyte and its possible underlying mechanism.

**Materials and methods:**

Chondrocytes obtained from rabbit articular cartilage were used in this study. The expression of zyxin, actin and vinculin, as well as the extracellular matrix (ECM) protein collagen type I, II and X (COL I, II and X) of chondrocytes were compared between zyxin‐knockdown group and negative control group, and between transforming growth factor‐β1 (TGF‐β1) treatment group and non‐treatment group, respectively.

**Results:**

Knockdown of zyxin increased the ratio of globular actin (G‐actin) to filamentous actin (F‐actin) of chondrocyte, which further inhibited expression of vinculin and chondrogenic marker COL II as well as hypertrophy marker COL X. On the other hand, chondrocytes treated with TGF‐β1 showed an enhanced expression of F‐actin, and a lower expression of zyxin compared to non‐treatment group. In response to TGF‐β1‐induced actin polymerization, expression of vinculin and COL I was increased, while expression of COL II and aggrecan was decreased.

**Conclusions:**

These results demonstrate supporting evidence that in chondrocytes the level of zyxin is closely associated with the state of actin polymerization. In particular, the change of zyxin and F‐actin parallels with the change of COL II and vinculin, respectively, indicating a major role of zyxin‐actin interaction in the synthesis of collagen ECM and the remodelling of cytoskeleton‐ECM adhesion.

## 1 INTRODUCTION

Articular cartilage is the highly specialized connective tissue of synovial joints. It is devoid of blood vessels and nerves and is kept healthy by cartilage producing cells called chondrocytes.^1^ The chondrocytes maintain a balance between cartilage matrix synthesis and degradation in a biomechanically dynamic environment.^2^ Once this balance is destroyed by an abnormal biological or mechanical stimulus to chondrocytes, the loss of cartilaginous tissue occurs, which finally leads to the degeneration of the whole joint.^3^ Therefore, the biomechanics and mechanobiology of articular chondrocytes is of great significance to articular cartilage homeostasis and pathology and has been under extensive investigation.^4^, ^5^ It is acknowledged that the biomechanical properties of chondrocytes are largely dependent on the cytoskeleton, in particular the actin cytoskeleton, as actin has been shown to regulate a series of chondrocyte characteristics including phenotype,^6^, ^7^ viscoelasticity,^8^, ^9^ stiffness^10^, ^11^ and responses to chemical/mechanical stimuli.^12^, ^13^

Like other eukaryotic cells, the actin cytoskeleton of chondrocyte is connected with the extracellular matrix (ECM) through focal adhesion. Zyxin is a LIM protein that concentrates at focal adhesions and is known to function as a mechanosensitive protein due to its ability to mobilize and relocate from focal adhesions to actin stress fibres in response to mechanical cues.^14^ Zyxin contains a number of dynamic structural features, including a proline‐rich domain that interacts with Ena/vasodilator‐stimulated phosphoprotein (VASP) family members, a nuclear export signal that allows translocation between the nucleus and cytoplasm, and three C‐terminal LIM domains involved in protein‐protein interactions.^15^ Recent studies show that zyxin could reinforce the actin cytoskeleton of human airway smooth muscle cells^16^ and could regulate endothelial von Willebrand factor secretion by reorganizing actin filaments around exocytic granules.^17^ However, the modulating role of zyxin on actin in chondrocytes has not been elucidated.

Vinculin, an actin‐binding protein considered to reinforce cell‐cell and cell‐matrix adhesions, is a component of both integrin‐ and cadherin‐based adhesion complexes and is recruited to both types of adhesions rapidly in response to mechanical cues through its interactions with talin and ɑ‐catenin, respectively.^18^, ^19^ Vinculin plays an important role in maintaining tissue integrity and is essential for persistent directional cell migration, indicating a role in forming a polarized connection between the actin cytoskeleton and adhesions.^20^ Moreover, studies have shown that vinculin and zyxin are colocalized at focal adhesions.^14^, ^21^ Therefore, vinculin might be involved in the interaction between actin and zyxin.

In this study, we investigated the modulating effects of zyxin on actin cytoskeleton and vinculin focal adhesion by constructing zyxin‐knockdown chondrocyte model. We performed immunofluorescence, qRT‐PCR and Western blot to measure actin and vinculin expression as well as collagen production of chondrocyte. Moreover, to study the influence of actin on zyxin and vinculin, we tested the changes of zyxin and vinculin when chondrocytes were treated with transforming growth factor‐β1 (TGF‐β1), as TGF‐β1 has been shown to mediate chondrocyte actin cytoskeleton through Ras homolog gene family, member A (RhoA) pathway.^22^, ^23^

## 2 MATERIALS AND METHODS

### 2.1 Chondrocytes isolation and culture

Primary chondrocytes were harvested according to a previous protocol.^24^ The New Zealand rabbits were purchased from the Third Military Medical University (Chongqing, China). The experiment was approved by Animal Care and Use Committee of the Third Military Medical University. Articular cartilage was removed from the knee joint of rabbits (1 month old, n = 3) with a sterile blade and minced. Cartilage pieces were digested with 0.2% w/v type collagenase (Sigma, St. Louis, MO, USA) in DMEM/F12 (Gibco, Grand Island, NY, USA) supplemented with 1% v/v penicillin‐streptomycin (Gibco) at 37°C in a 5% CO_2_ incubator overnight. Digested tissue was filtered with a 40‐μm‐cell strainer (BD Bioscience, Franklin Lakes, NJ, USA) followed by centrifugation at 400 ***g*** for 5 minutes and resuspended in DMEM/F12 supplemented with 10% v/v foetal bovine serum (Tbdscience, Tianjin, China) and 1% v/v penicillin‐streptomycin. Medium was changed every 3 days. Cells were passaged at 80%‐90% confluence, and the passage 2 (P2) cells were used in the following experiments. For human TGF‐β1 stimulation, chondrocytes were treated with 5 ng/mL TGF‐β1 (Peprotech, Rocky Hill, NJ, USA) for 24 hours.

### 2.2 siRNA transfection for zyxin knockdown

Non‐targeting SignalSilence Control siRNA (NC, sense, 5′‐UUCUCCGAACGUGUCACGUTT‐3′; antisense, 5′‐ACGUGACACGUUCGGAGAATT‐3′, Sangon Biotech, Shanghai, China) or zyxin siRNA (#627, sense, 5′‐GCACCGAAGCCCAAAGUAATT‐3′; antisense, 5′‐UUACUUUGGGCUUCGGUGCTT‐3′; #1009, sense, 5′‐CCAAGAAUGAUCCCUUCAATT‐3′; antisense, 5′‐UUGAAGGGAUCAUUCUUGGTT‐3′; #1448, sense, 5′‐ GGAAGCUGCUUCUUCCACUTT‐3′; antisense, 5′‐AGUGGAAGAAGCAGCUUCCTT‐3′, Sangon Biotech) were transfected into P2 chondrocytes by X‐tremeGene siRNA Transfection Reagent (Roche, Basel, Switzerland) according to the manufacturer's instruction. Approximately 2 × 10^5^ cells were transfected with 150 pmol of siRNA using X‐tremeGene siRNA Transfection Reagent. Briefly, 10 μL X‐tremeGene siRNA Transfection Reagent and 150 pmol of siRNA were diluted with 100 μL serum‐free Opti‐MEM I Medium (Gibco), respectively. Then, the complex was mixed and added to the cells, and the cells were returned to the incubator. To verify the effect of siRNA transfection, qRT‐PCR for zyxin gene expression was performed 24 hours after transfection, and Western blot for zyxin protein expression was performed 48 hours after transfection.

### 2.3 Quantitative real‐time PCR

Total RNA was extracted from cells using TRIzol reagent (Invitrogen, Carlsbad, CA, USA), and the RNA concentration was determined using a NanoDrop‐2000 spectrophotometer (Thermo Scientific, Wilmington, DE, USA). Then, the RNA was reverse transcribed to cDNA using Transcriptor cDNA Synth. kit 2 (Roche) according to the manufacturer's instruction. Quantitative real‐time PCR based on FS Essential DNA Green Master (Roche) was performed using primers specific for zyxin, vinculin, COL1A1, COL2A1, COL10A1 and GAPDH (Sangon Biotech). Primer sequences were as follows: zyxin forward, 5′‐CGAGAGCTGCTACACGGACA‐3′; zyxin reverse, 5′‐GCAGACCACACAGGTGAAGC‐3′; vinculin forward, 5′‐AAGCGGATTAGAACCAACCTCTTACAG‐3′; vinculin reverse, 5′‐GCTTCTCGCACAGTCTCCTTCAC‐3′; COL1A1 forward, 5′‐ CAATCACGCCTCTCAGAACA‐3′; COL1A1 reverse, 5′‐TCGGCAACAAGTTCAACATC‐3′; COL2A1 forward, 5′‐CAACAACCAGATCGAGAGCA‐3′; COL2A1 reverse, 5′‐CCAGTAGTCACCGCTCTTCC‐3′; COL10A1 forward, 5′‐GAATGGCACGCCTGTAATGT‐3′; COL10A1 reverse, 5′‐CCATTGGACTCAGCGTTAGG‐3′; GAPDH forward, 5′‐CGACATCAAGAAGGTGGTGA‐3′; GAPDH reverse, 5′‐ATCGAAGGTGGAGGAGTGG‐3′. Each reaction contained 5 μL cDNA, 10 μL FS Essential DNA Green master mix, 3 μL water (PCR grade) and 1 μL each of forward and reverse primers (10 μmol/L). Reactions were performed in triplicate. Examination of the melting curve for non‐specific peaks were performed to ensure specificity of PCR reactions, and mRNA levels were determined from Ct values according to a previously published method.^25^

### 2.4 Immunofluorescence

The cells were washed with PBS and fixed with 4% formaldehyde for 10 minutes at room temperature (RT). Then, the cells were washed three times with cold PBS and treated with 0.2% v/v Triton X‐100 in PBS for 10 minutes at RT. The cells were washed again three times with PBS and blocked 1 hour with 1% w/v BSA in PBST (0.1% v/v Tween‐20 in PBS) with 0.225% w/v glycine at RT followed by incubation with rabbit polyclonal antibody anti‐zyxin (Proteintech, Rosemont, IL, USA, 10330‐1‐AP), anti‐vinculin (Proteintech, 26520‐1‐AP), anti‐collagen type I (BIOYE, Shanghai, China, BYK‐0578R), anti‐collagen type II (BIOYE, BYK‐10589R) and anti‐collagen type X (BIOYE, BYK‐0554R) at the dilution of 1:50, 1:50, 1:300, 1:300 and 1:300, respectively in 1% w/v BSA in PBST overnight at 4°C. Next, the cells were washed three times with PBST and incubated with goat anti‐rabbit secondary antibody (ZSGB‐BIO, Beijing, China, ZF‐0511, 1:200) for 1 hour at RT. Afterward, the cells were washed three times, stained with Alexa Fluor^™^ 594 Phalloidin (Invitrogen) for 20 minutes at RT and DAPI (Biosharp, Beijing, China) for 5 minutes at 37°C, washed again and imaged with fluorescence microscopy. To quantify the fluorescent intensity, at least three staining images for each group were analyzed using ImageJ (NIH). The average fluorescence intensity of filamentous actin (F‐actin), vinculin, collagen type I, II and X (COL I, II and X) was determined by dividing the corresponding cell area with the optical density (OD).

### 2.5 Alcian blue staining

The cells were washed with PBS and fixed with 4% formaldehyde for 10 minutes at RT. Then, the cells were washed three times with PBS and stained with Alcian blue (Cyagen, Santa Clara, CA, USA) for 30 minutes. The cells were washed again three times with PBS and imaged with microscopy. Staining was quantified by solubilizing the sample in 6 mol/L guanidine hydrochloride for 8 hours at RT according to a published method.^26^ The OD value at 620 nm was measured by spectrophotometry.

### 2.6 Western blot

The cells were lysed in RIPA with PMSF (Beyotime, Shanghai, China) on ice for 5 minutes and removed with a scraper. Then the lysate was centrifuged at 16 100 ***g*** for 5 minutes, and the supernatant was collected. The protein concentration was determined by BCA Protein Assay Kit (Beyotime). The samples were diluted with SDS‐PAGE Sample Loading Buffer (Beyotime) and kept at 100°C for 10 minutes. 20 μg of protein was loaded in 10% SDS gel (CWBIO, Beijing, China) and run for 1 hour at 120 V followed by transferring onto a PVDF membrane for 1.5 hour at 200 mA. The membrane was washed twice with Milli‐Q water, stained with Ponceau S for protein visualization, and washed three times with 2% v/v TBST (tris‐buffered saline with Tween‐20). Then, the membrane was blocked with 5% w/v skimmed milk powder in TBST for 1 hour at RT, washed with TBST, and incubated with rabbit polyclonal antibody anti‐zyxin, anti‐vinculin, anti‐actin (Abcam, Cambridge, UK, ab69512), anti‐collagen type I, anti‐collagen type II, anti‐collagen type X and anti‐GAPDH (BIOYE, BYK‐2188R) diluted with Primary Antibody Dilution Buffer (Beyotime) at a dilution of 1:1000, 1:2000, 1:1000, 1:400, 1:400, 1:1000 and 1:5000, respectively overnight at 4°C shaker. Next, the membrane was washed four times with TBST for 10 minutes, incubated with the goat anti‐rabbit secondary antibody (ZSGB‐BIO, ZB‐2301, 1:10 000) for 1 hour at RT, washed again four times, and visualized with Western ECL Substrate (Thermo Scientific) for chemiluminescence.

### 2.7 Actin quantification

Quantification of F‐actin to globular actin (G‐actin) ratio was also performed by Western blot according to a previously published method.^27^, ^28^ The cells were lysed in 150 μL of extraction buffer (0.1% Triton X‐100 in PBS and complete protease inhibitor) and removed with a scraper. Then the lysate was centrifuged at 16 100 ***g*** for 5 minutes. The supernatant was used to measure soluble actin (G‐actin). The precipitate was resuspended in 150 μL of RIPA buffer and used to measure filamentous actin (as a reflection of insoluble F‐actin). Equal volume of samples from the supernatant (G‐actin) and precipitate (F‐actin) were loaded and analyzed by Western blot.

### 2.8 Statistical analysis

Data were represented as mean ± standard deviation (SD) and obtained from three independent experiments. One‐way analysis of variance (ANOVA) was performed for statistical analysis between control group (NC) and experiment groups (mostly #1009) using spss statistics 20 (IBM, Chicago, IL, USA) software. Mann‐Whitney *U* test was performed for statistical analysis when the data didn't accord with the homogeneity of variance. *P* values less than 0.05 were considered as statistically significant.

## 3 RESULTS

### 3.1 siRNA transfection for zyxin knockdown

**Figure 1:**
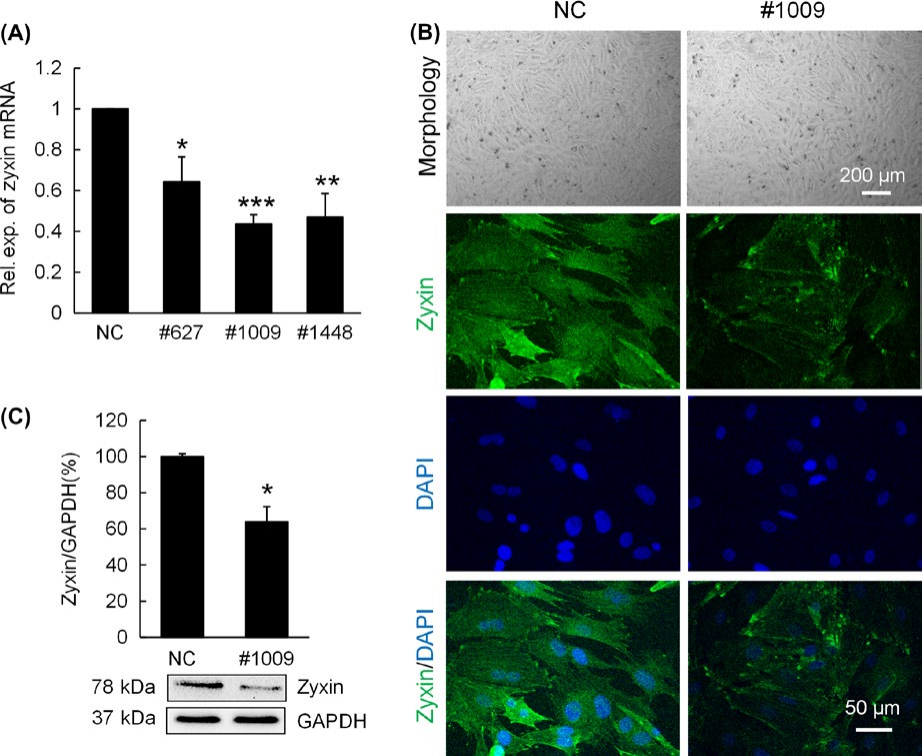
siRNA transfection for zyxin knockdown in rabbit chondrocytes. (A) mRNA expression of zyxin in chondrocytes transfected with non‐targeting SignalSilence Control siRNA (NC) and zyxin siRNA (#627, #1009, and #1448). Data were normalized to NC; *P < 0.05, **P < 0.01, ***P < 0.001. (B) Cell morphology and immunofluorescence imaging of zyxin (green) of chondrocytes transfected with NC and siRNA #1009. (C) Western blot analysis of zyxin of chondrocytes transfected with NC and siRNA #1009; *P < 0.05

Non‐targeting SignalSilence Control siRNA (NC) or zyxin siRNA (#627, #1009, #1448) were transfected into P2 chondrocytes. Expression of zyxin was assessed. Zyxin gene expression in chondrocytes transfected with zyxin siRNA #627, #1009 and #1448 was 64.28 ± 12.28% 43.68 ± 4.5% and 47.13 ± 11.4%, respectively of zyxin gene expression in chondrocytes transfected with NC (Figure 1A). Among these siRNA, #1009 resulted in a best knockdown of zyxin gene and was used in the following experiments. Chondrocytes transfected with #1009 siRNA showed a similar morphology but a weaker zyxin staining compared to NC group (Figure 1B). In line with the gene expression data, zyxin protein expression in chondrocytes transfected with #1009 siRNA was also much lower (63.98 ± 8.38%) than NC group (Figure 1C).

### 3.2 Zyxin knockdown impairs F‐actin of chondrocyte

**Figure 2:**
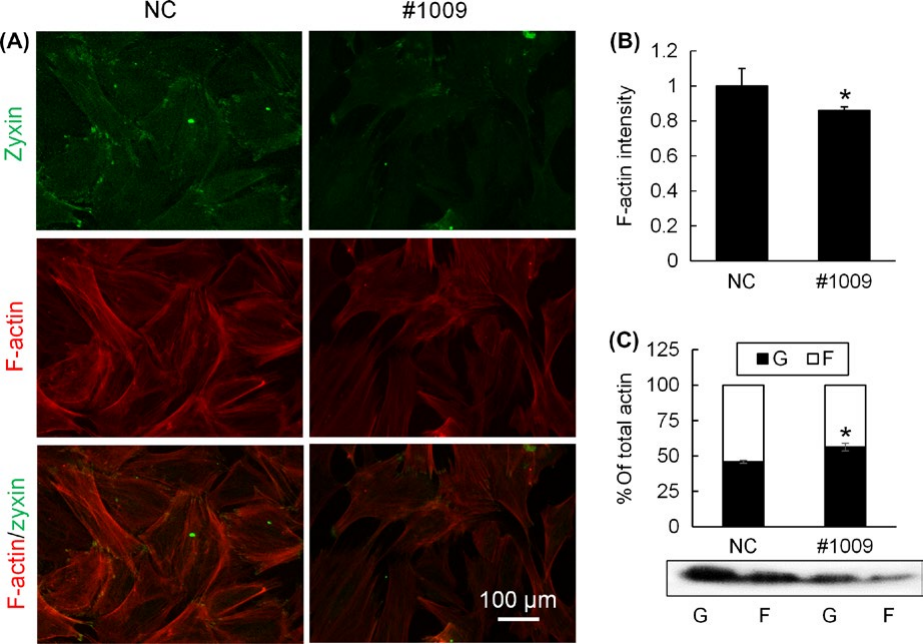
Zyxin knockdown affects actin cytoskeleton of chondrocyte. (A) Immunofluorescence imaging of zyxin (green) and F‐actin (red) of chondrocytes transfected with NC and siRNA #1009. (B) Quantification of F‐actin fluorescence intensity. Data were normalized to NC; *P < 0.05 (Mann‐Whitney *U* test). (C) Western blot analysis of G‐actin and F‐actin of chondrocytes transfected with NC and siRNA #1009; *P < 0.05

After zyxin knockdown, chondrocytes were double‐stained with zyxin and F‐actin (Figure 2A). #1009 group (zyxin knockdown) had fewer actin stress fibres and a slightly lower F‐actin intensity (Figure 2B) than NC group, both indicating a minor impairment of actin cytoskeleton in #1009 group. This was confirmed by Western blot analysis: in NC group, ~45% of actin was soluble G‐actin and ~55% of actin was filamentous F‐actin; while in #1009 group, ~55% of actin was G‐actin and ~45% was F‐actin (Figure 2C). All these results indicated that zyxin knockdown impaired the actin cytoskeleton, as a fraction of F‐actin disorganized into soluble G‐actin.

### 3.3 Zyxin knockdown reduces vinculin expression of chondrocyte

**Figure 3:**
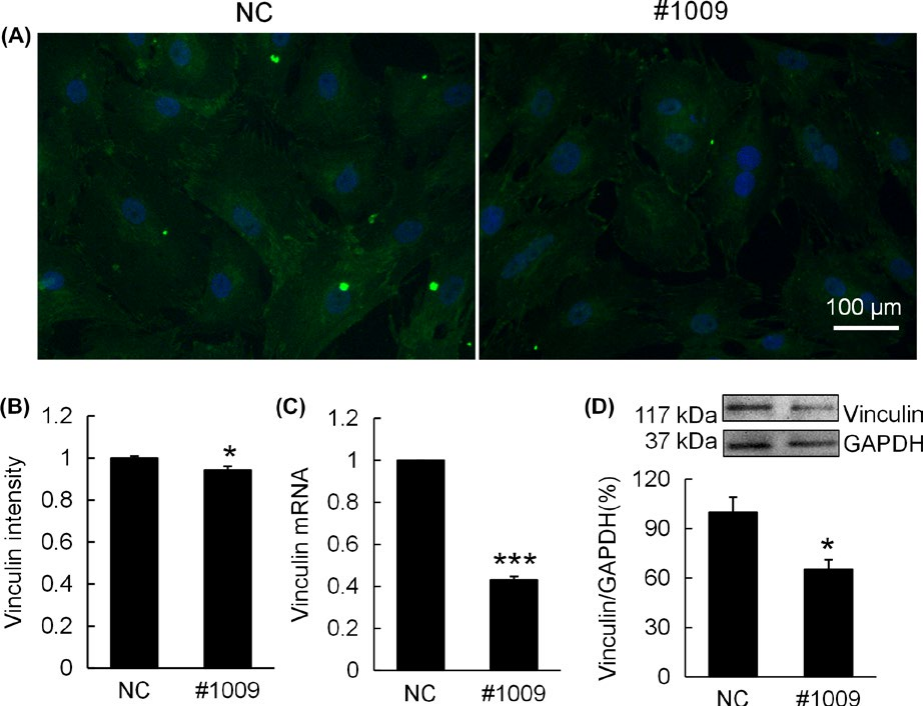
Zyxin knockdown reduces vinculin expression of chondrocyte. (A) Immunofluorescence imaging of vinculin (green) of chondrocytes transfected with NC and siRNA #1009. (B) Quantification of vinculin fluorescence intensity. Data were normalized to NC; *P < 0.05. (C) mRNA expression of vinculin of chondrocytes transfected with NC and siRNA #1009. Data were normalized to NC; ***P < 0.001. (D) Western blot analysis of vinculin of chondrocytes transfected with NC and siRNA #1009; *P < 0.05

After observing the role zyxin plays in maintenance of actin cytoskeleton, we investigated the effect of zyxin knockdown on the expression of focal adhesion protein vinculin. Immunofluorescence staining showed that vinculin deposition in #1009 group was more concentrated at cell edge compared to that in NC group (Figure 3A), and vinculin intensity in #1009 group was slightly lower (Figure 3B). PCR results showed that vinculin gene expression was decreased to 43.23 ± 1.48% in response to zyxin knockdown (Figure 3C). Similarly, Western blot analysis demonstrated that vinculin deposition in chondrocytes reduced to 65.24 ± 5.94% after zyxin knockdown (Figure 3D).

### 3.4 Zyxin knockdown affects collagen production of chondrocyte

**Figure 4:**
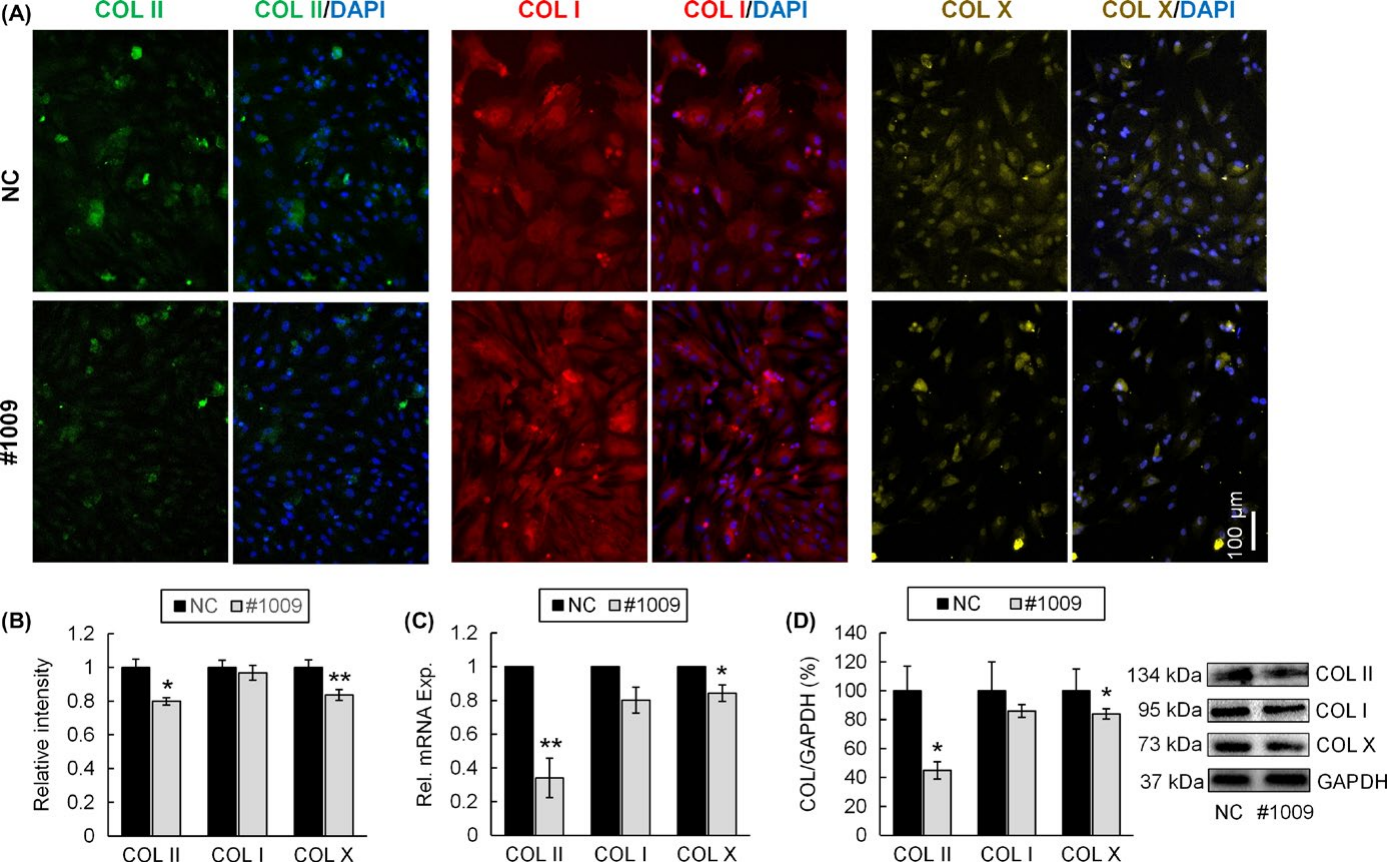
Zyxin knockdown affects collagen expression of chondrocytes. (A) Immunofluorescence imaging of COL II (green), COL I (red) and COL X (yellow) of chondrocytes transfected with NC and siRNA #1009. (B) Quantification of COL II, COL I and COL X fluorescence intensity. Data were normalized to NC; *P < 0.05, **P < 0.01. (C) mRNA expression of COL II, COL I and COL X of chondrocytes transfected with NC and siRNA #1009. Data were normalized to NC; *P < 0.05, **P < 0.01. (D) Western blot analysis of COL II, COL I and COL X of chondrocytes transfected with NC and siRNA #1009. *P < 0.05 (COL X group data were analyzed by Mann‐Whitney *U* test)

We next monitored the expression of chondrogenic marker COL II, de‐differentiation marker COL I and hypertrophic marker COL X. Immunofluorescence staining showed that COL II and COL X deposition in chondrocytes of #1009 group was weaker than that of NC group (Figure 4A,B). In line with the staining results, COL II and COL X gene expression decreased to 34.04 ± 11.61% and 84.32 ± 4.95%, respectively in response to zyxin knockdown (Figure 4C). In line with the gene expression data, Western blot analysis also showed that COL II and COL X deposition in chondrocytes reduced to 44.92 ± 6.06% and 86.07 ± 4.47%, respectively after zyxin knockdown (Figure 4D). There was no difference in COL I expression between the two groups at either gene or protein level.

### 3.5 TGF‐β1 increases F‐actin and vinculin expression but reduces zyxin expression, which in turn affects chondrocyte differentiation status

**Figure 5:**
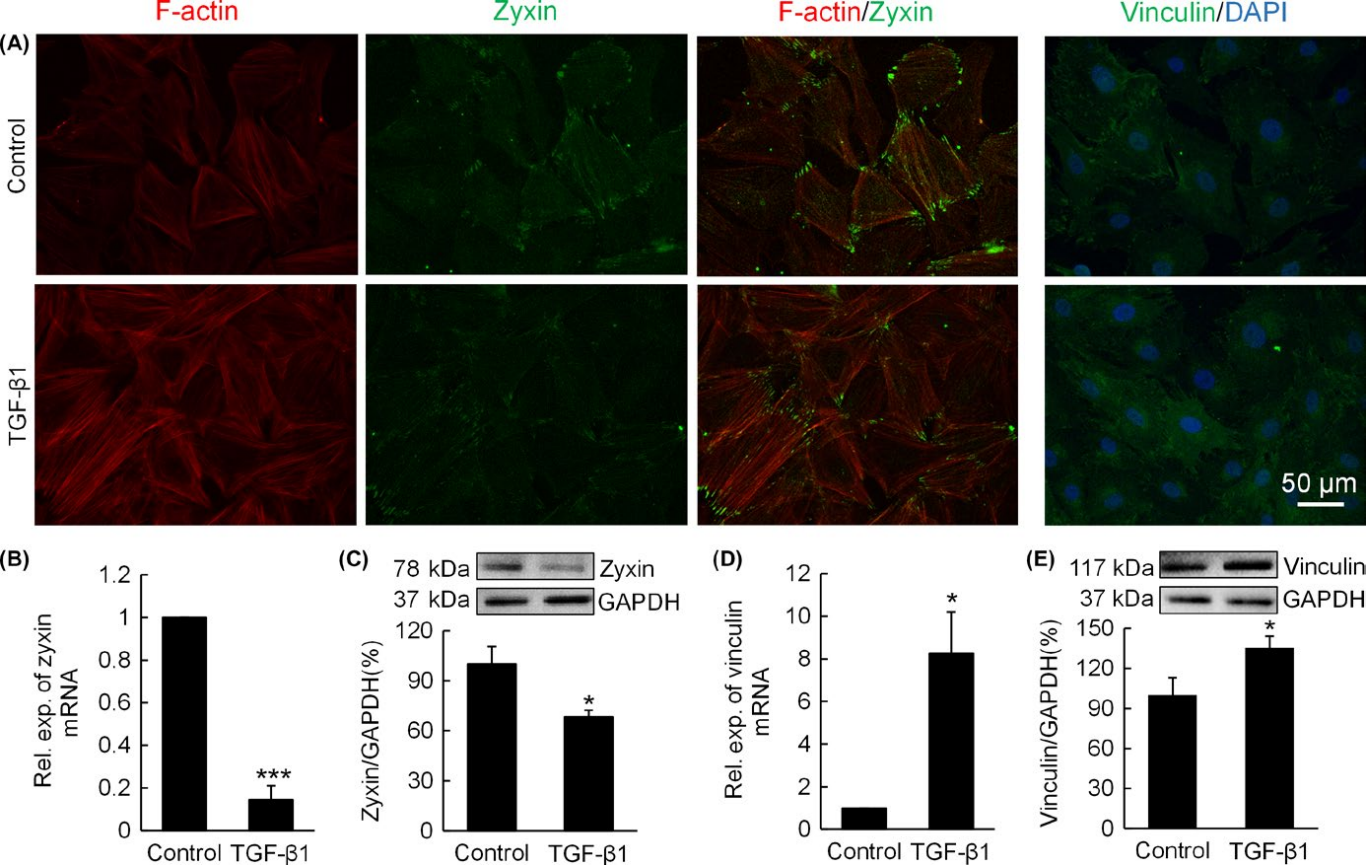
Transforming growth factor‐β1 (TGF‐β1) treatment increases F‐actin and vinculin expression of chondrocyte, but reduces zyxin expression. (A) F‐actin, zyxin and vinculin staining of chondrocytes treated with TGF‐β1. (B) mRNA expression of zyxin of chondrocytes treated with TGF‐β1. Data were normalized to control; ***P < 0.001. (C) Western blot analysis of zyxin of chondrocytes treated with TGF‐β1; *P < 0.05. (D) mRNA expression of vinculin of chondrocytes treated with TGF‐β1. Data were normalized to control; *P < 0.05. (E) Western blot analysis of vinculin of chondrocytes treated with TGF‐β1; *P < 0.05

After observing the important role zyxin plays in maintenance of actin cytoskeleton and collagen matrix, we treated the chondrocytes with TGF‐β1. Phalloidin staining demonstrated that the F‐actin deposition in chondrocytes increased, and the formation of stress fibres was enhanced; zyxin staining showed that in control group zyxin was located at the cell circumference; while in TGF‐β1 treatment group, zyxin was more located at cell ends; for vinculin staining, TGF‐β1 treatment group showed a higher fluorescent intensity than control group (Figure 5A). Zyxin gene expression showed an approximate 85% decrease in response to TGF‐β1 treatment (Figure 5B). Likewise, Western blot analysis of zyxin demonstrated that zyxin deposition in chondrocytes decreased after TGF‐β1 treatment (Figure 5C). On the contrary, vinculin gene expression increased by approximately 7 fold after TGF‐β1 treatment (Figure 5D), and Western blot analysis showed that vinculin deposition at protein level also increased by 35% after TGF‐β1 treatment (Figure 5E).

**Figure 6:**
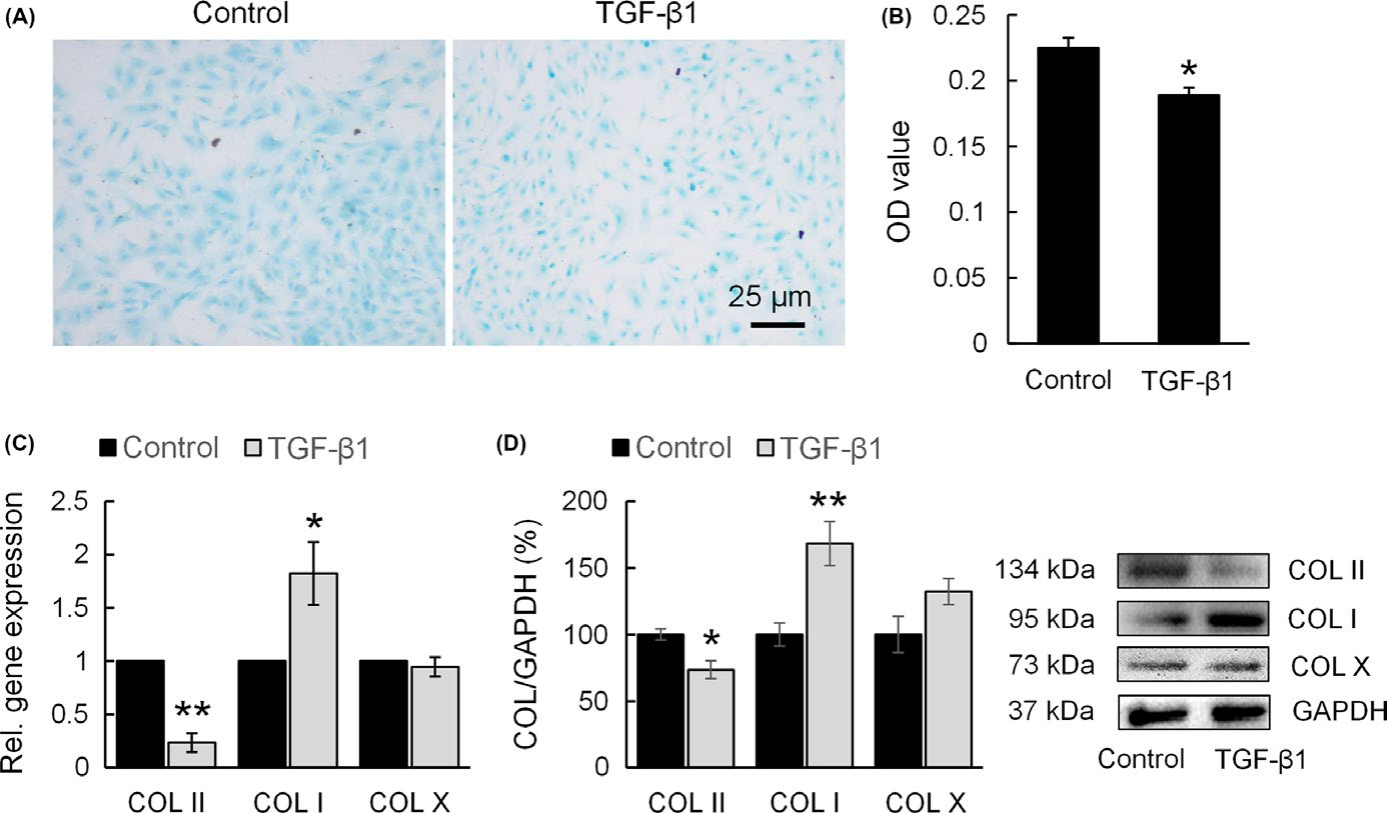
Transforming growth factor‐β1 (TGF‐β1) treatment affects ECM synthesis of chondrocyte. (A) Alcian blue staining of chondrocytes treated with TGF‐β1. (B) Alcian blue staining quantification by OD value at 620 nm; *P < 0.05. (C) mRNA expression of COL I, COL II and COL X of chondrocytes treated with TGF‐β1. Data were normalized to control; *P < 0.05, **P < 0.01. (D) Western blot analysis of COL I, COL II and COL X of chondrocytes treated with TGF‐β1; *P < 0.05, **P < 0.01

Since TGF‐β1 treatment down‐regulated the expression of zyxin, we therefore speculated that TGF‐β1‐treated chondrocytes had the same phenotypic change as zyxin knock‐down chondrocytes. Alcian blue staining showed that the aggrecan (ACAN) deposition in chondrocytes decreased in response to TGF‐β1 treatment (Figure 6A,B). COL II gene expression showed approximately a 75% decrease, while COL I gene expression showed approximately an 80% increase in response to TGF‐β1 treatment (Figure 6C). Western blot analysis further confirmed that COL II deposition decreased, and COL I deposition increased in chondrocytes after TGF‐β1 treatment (Figure 6D).

## 4 DISCUSSION

The major findings of this study were (a) zyxin knockdown of chondrocytes resulted in a lower expression of actin cytoskeleton and vinculin focal adhesion, as well as a declining production of COL II and COL X; (b) TGF‐β1 treatment induced a reinforcement of actin cytoskeleton along with vinculin and COL I upregulation and a marked decline of COL II expression. These findings indicate that in chondrocytes the zyxin‐involved actin regulation has a major impact on collagen ECM synthesis of chondrocytes.

In our study, the zyxin‐knockdown‐induced changes of actin cytoskeleton were evidenced by reduced stress fibres and increased G/F‐actin ratio (Figure 2). It has been shown in other cell types that zyxin recruits actin polymerization factors such as VASP and actin crosslinkers such as alpha‐actinin, these factors are essential to ensure the assembly of actin bundles into stress fibres.^29^, ^30^ This could explain our finding in chondrocytes that zyxin knockdown weakens actin polymerization. To examine whether alteration of actin cytoskeleton leads to corresponding changes of chondrocyte focal adhesion, we measured the expression of vinculin—a membrane‐cytoskeletal protein in focal adhesion plaques that are involved in linkage of integrin adhesion molecules to the actin cytoskeleton. Interestingly, we found that vinculin expression of chondrocyte lowered as a result of the increased G/F‐actin ratio in response to zyxin knockdown (Figure 3), but rose as a result of F‐actin enhancement in response to TGF‐β1 treatment (Figure 5A,D,E). The change of vinculin expression paralleled with the change of F‐actin expression, indicating a matched cytoskeletal stiffness (as determined by F‐actin) against cellular contractile strength (as determined by focal adhesion).

We next investigated whether zyxin knockdown could affect chondrocyte differentiation by measuring the ECM proteins COL II (chondrogenic marker), COL I (de‐differentiation marker) and COL X (hypertrophic marker). Our results demonstrated that zyxin knockdown led to a lower expression of both COL II and COL X (Figure 4), which could be attributed to the increased G/F‐actin ratio induced by zyxin‐knockdown. Similarly, it has been shown recently that disassembly of the actin cytoskeleton results in impairment of TGF‐β pathway, which controls collagen production in fibroblasts. Cytoskeleton disassembly rapidly down‐regulates TGF‐β type II receptor levels, and this down‐regulation leads to reduced activation of downstream effectors Smad2/Smad3 and connective tissue growth factor, resulting in a decreased collagen production.^31^

After observing the effects of zyxin‐knockdown on chondrocyte actin cytoskeleton and differentiation, we treated normal chondrocytes with TGF‐β1. It has been shown that TGF‐β induces a rapid reorganization of the actin cytoskeleton and results in the formation of stress fibres with the activation of the Rho family of GTPases: ras‐related C3 botulinum toxin substrate 1 (Rac), cell division control protein 42 homolog (CDC42) or RhoA in a series of cell types.^32^, ^33^, ^34^ In chondrocytes, the studies of TGF‐β signalling are mostly focused on cartilage pathophysiology,^35^, ^36^ with very few studies on actin dynamics. Leipzig et al^37^ found that treatment of 5 ng/mL TGF‐β1 increased F‐actin levels in bovine articular, and ascribed this change to signalling crosstalk between growth factor receptors and focal adhesion complexes. Similarly, our results showed that treatment of 5 ng/mL TGF‐β1 promoted the formation of actin stress fibres, along with the upregulation of vinculin focal adhesion. Moreover, on account of F‐actin increase, the expression of actin polymerization regulator zyxin was inhibited, possibly due to a feedback regulation triggered by remodelling of the F‐actin cytoskeleton. However, Mise et al. found that zyxin promoter is a direct target of Smad3 after TGF‐β1 treatment in A549 cells, and activation of Smad3 upon TGF‐β1 treatment resulted in prominent zyxin expression.^38^ This finding is contrary to our results here, which might be due to the different local activities of TGF‐β1 within these two cell types.

**Figure 7:**
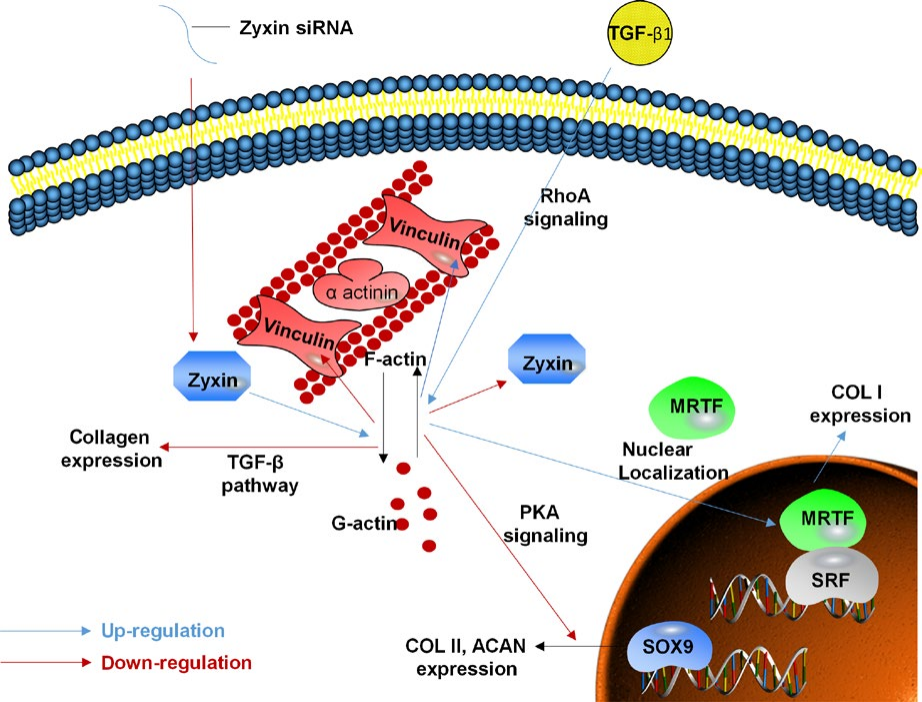
Schematic of zyxin‐based regulation mechanism of actin cytoskeleton and differentiation status of chondrocyte. Zyxin expression and actin polymerization state have a bidirectional relationship and mutually adjust differentiation of chondrocyte. MRTF: myocardin‐related transcription factor; PKA signalling: protein kinase A signalling; SRF: serum response factor

Since TGF‐β1 treatment down‐regulated the expression of zyxin, we hypothesized that chondrocytes had the same phenotypic changes in response to TGF‐β1 stimulation as to zyxin knockdown. Indeed, the GAG deposition and expression of COL II both decreased after TGF‐β1 treatment (Figure 6). The reason could be that the enhanced actin polymerization by TGF‐β1 stimulation, down‐regulates SOX9 expression through protein kinase A signalling (Figure 7).^39^, ^40^ It is noteworthy that the expression of COL I increased in response to TGF‐β1 treatment. This might be owing to the nuclear localization of myocardin‐related transcription factor (MRTF)—a G‐actin‐binding protein that has the ability to regulate COL I gene expression.^27^, ^28^ Here, the TGF‐β1 treatment reinforced actin polymerization, and the polymerization state has been shown to regulate the actin‐binding proteins into and out of the nucleus.^41^ In particular, nuclear localization of MRTF allows it to associate and act as a transcriptional co‐activator to serum response factor on promoter regions,^42^, therefore, enhancing COL I gene expression (Figure 7).

Overall, there are two limitations of this study. First, a zyxin‐knockdown model was used, which is inferior to zyxin‐knockout model. Second, the chondrocytes were cultured in a 2D environment, which could induce de‐differentiation and interfere with phenotype analysis. Nevertheless, our study demonstrated that the zyxin‐involved actin regulation has an impact on vinculin focal adhesion and collagen matrix of chondrocytes. When zyxin level is lower than normal, the expression of COL II was mainly affected; when F‐actin level is higher than normal, the expression of COL I was largely increased, and the expression of vinculin parallels with F‐actin. In conclusion, our results revealed the important role of zyxin‐involved actin regulation in vinculin focal adhesion and collagen matrix of chondrocytes.

## CONFLICT OF INTEREST

The authors declare no conflict of interest.
